# Bis(guanidinium) tris­(pyridine-2,6-dicarboxyl­ato-κ^3^ *O* ^2^,*N*,*O* ^6^)zirconate(II) tetra­hydrate

**DOI:** 10.1107/S1600536812011439

**Published:** 2012-03-24

**Authors:** Masoumeh Tabatabaee, Mahnaz Adineh, Zohreh Derikvand, Jafar Attar Gharamaleki

**Affiliations:** a Department of Chemistry, Yazd Branch, Islamic Azad University, Yazd, Iran; b Department of Chemistry, Faculty of Science, Khorramabad Branch, Islamic Azad, University, Khorramabad, Iran; c Faculty of Chemistry, Tarbiat Moallem University, 49 Mofateh Avenue, Tehran, Iran

## Abstract

In the title complex, (CH_6_N_3_)_2_[Zr(C_7_H_3_NO_4_)_3_]·4H_2_O, the Zr^IV^ ion lies on a twofold rotation axes and is coordinated by six O and three N atoms of three tridentate pyridine-2,6-dicarboxyl­ate ligands, forming a slightly distorted tricapped trigonal–prismatic geometry. In the crystal, O—H⋯O and N—H⋯O hydrogen bonds link the components into a three-dimensional network.

## Related literature

For related structures, see: Aghabozorg *et al.* (2005 ▶); Tabatabaee (2010 ▶); Tabatabaee *et al.* (2009 ▶, 2011*a* ▶,*b* ▶,*c* ▶, 2012 ▶); Derikvand *et al.* (2010 ▶); Attar Gharamaleki *et al.* (2009 ▶).
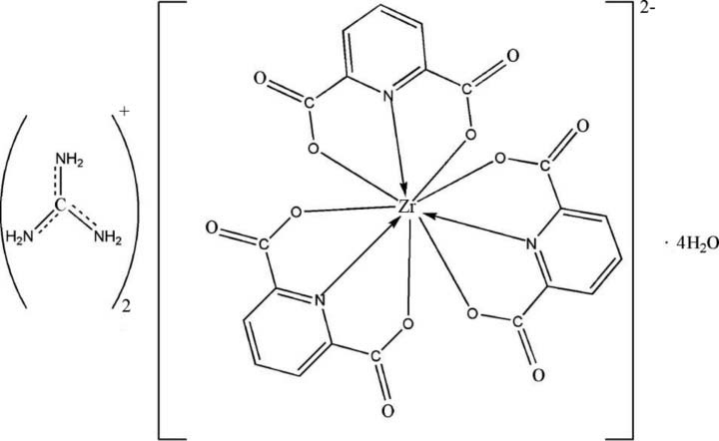


## Experimental

### 

#### Crystal data

(CH_6_N_3_)_2_[Zr(C_7_H_3_NO_4_)_3_]·4H_2_O
*M* *_r_* = 778.77
Orthorhombic, 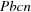

*a* = 17.2444 (9) Å
*b* = 10.8583 (5) Å
*c* = 16.5268 (8) Å
*V* = 3094.6 (3) Å^3^
*Z* = 4
Mo *K*α radiation
μ = 0.45 mm^−1^
*T* = 120 K
0.17 × 0.15 × 0.07 mm

#### Data collection

Bruker SMART 1000 CCD diffractometer
Absorption correction: multi-scan (*SADABS*; Bruker, 1998 ▶) *T*_min_ = 0.884, *T*_max_ = 0.970
32229 measured reflections
4116 independent reflections
2884 reflections with *I* > 2σ(*I*)
*R*_int_ = 0.068

#### Refinement

*R*[*F* ^2^ > 2σ(*F* ^2^)] = 0.056
*wR*(*F* ^2^) = 0.146
*S* = 1.07
4116 reflections
223 parameters
H-atom parameters constrained
Δρ_max_ = 0.87 e Å^−3^
Δρ_min_ = −0.47 e Å^−3^

### 

Data collection: *SMART* (Bruker, 1998 ▶); cell refinement: *SAINT* (Bruker, 1998 ▶); data reduction: *SAINT*; program(s) used to solve structure: *SHELXTL* (Sheldrick, 2008 ▶); program(s) used to refine structure: *SHELXTL*; molecular graphics: *SHELXTL*, *DIAMOND* (Brandenburg, 1999 ▶) and *Mercury* (Macrae *et al.*, 2006 ▶); software used to prepare material for publication: *SHELXTL*.

## Supplementary Material

Crystal structure: contains datablock(s) I, global. DOI: 10.1107/S1600536812011439/lh5430sup1.cif

Structure factors: contains datablock(s) I. DOI: 10.1107/S1600536812011439/lh5430Isup2.hkl

Additional supplementary materials:  crystallographic information; 3D view; checkCIF report

## Figures and Tables

**Table 1:** Hydrogen-bond geometry (Å, °)

*D*—H⋯*A*	*D*—H	H⋯*A*	*D*⋯*A*	*D*—H⋯*A*
N3—H3*NA*⋯O1	0.77	2.23	3.003 (4)	175
N3—H3*NB*⋯O2*W*^i^	0.86	2.15	2.930 (4)	150
N4—H4*NA*⋯O2*W*^i^	0.83	2.04	2.818 (4)	156
N4—H4*NB*⋯O1*W*^ii^	0.78	2.06	2.834 (4)	175
N5—H5*NA*⋯O2	0.88	1.95	2.828 (4)	171
N5—H5*NB*⋯O3^iii^	0.79	2.45	3.143 (4)	148
N5—H5*NB*⋯O4^iii^	0.79	2.52	3.171 (4)	141
O1*W*—H1*WA*⋯O3^iv^	0.84	2.33	3.041 (3)	143
O1*W*—H1*WA*⋯O5^iv^	0.84	2.38	3.076 (3)	140
O1*W*—H1*WB*⋯O6	0.89	1.87	2.761 (3)	175
O2*W*—H2*WA*⋯O5^iv^	0.86	2.07	2.909 (3)	165
O2*W*—H2*WA*⋯O6^iv^	0.86	2.57	3.085 (3)	119
O2*W*—H2*WB*⋯O4^v^	0.94	1.84	2.745 (3)	160

Symmetry codes: (i) 
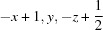
; (ii) 
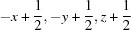
; (iii) 
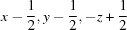
; (iv) 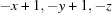; (v) 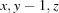.

**Fig. 1.**
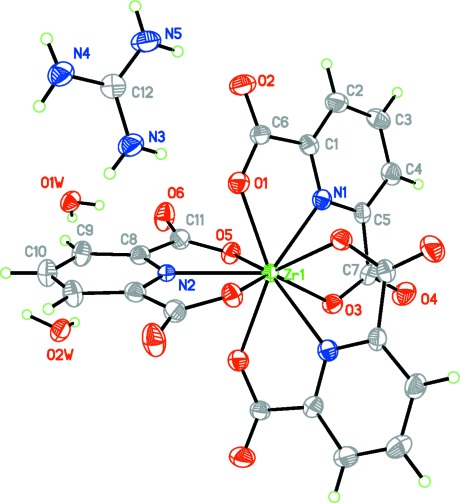
The molecular structure of (I) with ellipsoids drawn at the 50% probability level. The unlabeled atoms are related by the symmetry operator (-x+1, y, -z+1/2). Only the symmetry unique anions and water molecules are shown.

**Fig. 2.**
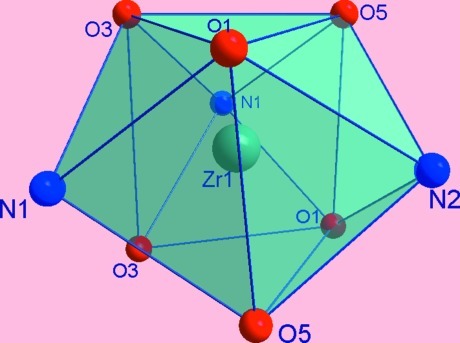
View of the coordination environment of the ZrIV ion.

**Fig. 3.**
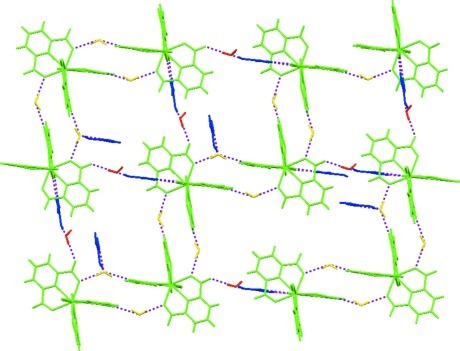
Part of the crystal structure of (I). The donor to acceptor distances of the hydrogen bonds are shown as dotted lines.

## supplementary crystallographic information

### Comment

In recent years, our research group has been interested in the synthesis of proton transfer compounds and study of their behavior with metal ions. We have focused on the proton delivery of polycarboxylic acids. Pyridine-2,6-dicarboxylic acid (pydcH_2_) is a very important carboxylate derivative and has attracted much interest in coordination chemistry. This is the acid we have utilized widely in our studies (Tabatabaee *et al.*, 2010, 2011*a*, 2011*b*, 2011*c*, 2012; Derikvand *et al.*, 2010; Attar Gharamaleki *et al.*, 2009). In this paper we report the crystal structure of the title complex (I). The Zr^IV^ ion lies on a twofold rotation axis. The asymmetric unit and the symmetry complete cation is shown in Fig. 1. The Zr^IV^ atom is coordinated by three tridentate pydc ligands forming a slightly distorted tricapped trigonal prismatic environment (Fig. 2). The Zr—N distances and Zr—O distances are consistent with those found in (pydaH)_2_[Zr(pydc)_3_].5H_2_O (Aghabozorg *et al.*, 2005). In the crystal, O—H···O and N—H···O hydrogen bonds (Table 1) link the components into a three-dimensional network (Fig. 3).

### Experimental

An aqueous solution of ZrOCl_2_.8H_2_O, (161 mg, 0.5 mmol) in water (5 ml) was added to a stirring solution of (20 ml) pyridine-2,6-dicarboxylic acid (176 mg, 1 mmol) and guanidine hydrochloride (95 mg, 1 mmol). The reaction mixture was stirred at 298K for 1 h. Colorless crystals of the title compound were obtained after 4 days by slow evaporation of the solvent at room temperature.

### Refinement

H atoms bonded to C atoms were placed in calculated positions. The H atoms of water molecules and NH_2_ groups were located in difference Fourier maps and included in 'as found' positions. All hydrogen atoms were refined in isotropic approximatiom in a riding-model approximation with *U*_iso_(H) parameters equal to 1.2 *U*_eq_(C), 1.5 *U*_eq_(O,*N*).

### Crystal data

**Table:**

(CH_6_N_3_)_2_[Zr(C_7_H_3_NO_4_)_3_]·4H_2_O	*F*(000) = 1592
*M**_r_* = 778.77	*D*_x_ = 1.672 Mg m^−^^3^
Orthorhombic, *P**b**c**n*	Mo *K*α radiation, λ = 0.71073 Å
Hall symbol: -P 2n 2ab	Cell parameters from 4151 reflections
*a* = 17.2444 (9) Å	θ = 2.3–26.0°
*b* = 10.8583 (5) Å	µ = 0.45 mm^−^^1^
*c* = 16.5268 (8) Å	*T* = 120 K
*V* = 3094.6 (3) Å^3^	Prism, colorless
*Z* = 4	0.17 × 0.15 × 0.07 mm

### Data collection

**Table:**

Bruker SMART 1000 CCD diffractometer	4116 independent reflections
Radiation source: normal-focus sealed tube	2884 reflections with *I* > 2σ(*I*)
Graphite monochromator	*R*_int_ = 0.068
ω scans	θ_max_ = 29.0°, θ_min_ = 2.2°
Absorption correction: multi-scan (*SADABS*; Bruker, 1998)	*h* = −23→23
*T*_min_ = 0.884, *T*_max_ = 0.970	*k* = −14→14
32229 measured reflections	*l* = −22→22

### Refinement

**Table:**

Refinement on *F*^2^	Primary atom site location: structure-invariant direct methods
Least-squares matrix: full	Secondary atom site location: difference Fourier map
*R*[*F*^2^ > 2σ(*F*^2^)] = 0.056	Hydrogen site location: mixed
*wR*(*F*^2^) = 0.146	H-atom parameters constrained
*S* = 1.07	*w* = 1/[σ^2^(*F*_o_^2^) + (0.0562*P*)^2^ + 8.430*P*] where *P* = (*F*_o_^2^ + 2*F*_c_^2^)/3
4116 reflections	(Δ/σ)_max_ < 0.001
223 parameters	Δρ_max_ = 0.87 e Å^−^^3^
0 restraints	Δρ_min_ = −0.47 e Å^−^^3^

### Special details

**Table:**

Geometry. All e.s.d.'s (except the e.s.d. in the dihedral angle between two l.s. planes) are estimated using the full covariance matrix. The cell e.s.d.'s are taken into account individually in the estimation of e.s.d.'s in distances, angles and torsion angles; correlations between e.s.d.'s in cell parameters are only used when they are defined by crystal symmetry. An approximate (isotropic) treatment of cell e.s.d.'s is used for estimating e.s.d.'s involving l.s. planes.
Refinement. Refinement of *F*^2^ against ALL reflections. The weighted *R*-factor *wR* and goodness of fit *S* are based on *F*^2^, conventional *R*-factors *R* are based on *F*, with *F* set to zero for negative *F*^2^. The threshold expression of *F*^2^ > σ(*F*^2^) is used only for calculating *R*-factors(gt) *etc*. and is not relevant to the choice of reflections for refinement. *R*-factors based on *F*^2^ are statistically about twice as large as those based on *F*, and *R*- factors based on ALL data will be even larger.

### Fractional atomic coordinates and isotropic or equivalent isotropic displacement parameters (Å^2^)

**Table:**

	*x*	*y*	*z*	*U*_iso_*/*U*_eq_	
Zr1	0.5000	0.72625 (4)	0.2500	0.01631 (13)	
O1	0.38154 (13)	0.6592 (2)	0.28200 (14)	0.0245 (5)	
O2	0.25204 (15)	0.6675 (3)	0.27362 (17)	0.0349 (6)	
O3	0.54360 (12)	0.8668 (2)	0.16530 (13)	0.0226 (5)	
O4	0.53142 (16)	1.0370 (2)	0.09047 (17)	0.0362 (6)	
O5	0.47334 (14)	0.6443 (2)	0.12914 (14)	0.0236 (5)	
O6	0.42065 (17)	0.4926 (2)	0.05627 (15)	0.0354 (6)	
N1	0.40034 (15)	0.8418 (2)	0.18731 (16)	0.0214 (5)	
N2	0.5000	0.5072 (3)	0.2500	0.0171 (7)	
C1	0.32630 (19)	0.8123 (3)	0.20019 (19)	0.0225 (6)	
C2	0.2655 (2)	0.8764 (3)	0.1641 (2)	0.0292 (7)	
H2A	0.2131	0.8533	0.1731	0.035*	
C3	0.2842 (2)	0.9750 (3)	0.1146 (2)	0.0295 (7)	
H3A	0.2441	1.0216	0.0898	0.035*	
C4	0.3609 (2)	1.0061 (3)	0.1010 (2)	0.0273 (7)	
H4A	0.3743	1.0733	0.0670	0.033*	
C5	0.41742 (19)	0.9359 (3)	0.13861 (19)	0.0219 (6)	
C6	0.31649 (18)	0.7054 (3)	0.25566 (19)	0.0218 (6)	
C7	0.50369 (18)	0.9516 (3)	0.12932 (18)	0.0205 (6)	
C8	0.47612 (18)	0.4462 (3)	0.18438 (19)	0.0222 (6)	
C9	0.4742 (2)	0.3187 (3)	0.1823 (2)	0.0273 (7)	
H9A	0.4557	0.2761	0.1360	0.033*	
C10	0.5000	0.2551 (4)	0.2500	0.0284 (10)	
H10A	0.5000	0.1676	0.2500	0.034*	
C11	0.45375 (19)	0.5307 (3)	0.1163 (2)	0.0240 (6)	
N3	0.33875 (17)	0.4432 (3)	0.38628 (19)	0.0312 (7)	
H3NA	0.3482	0.5016	0.3616	0.047*	
H3NB	0.3752	0.4039	0.4112	0.047*	
N4	0.25724 (18)	0.3150 (3)	0.45574 (18)	0.0314 (7)	
H4NA	0.2971	0.2749	0.4652	0.047*	
H4NB	0.2158	0.2885	0.4626	0.047*	
N5	0.20814 (17)	0.4727 (3)	0.3776 (2)	0.0364 (8)	
H5NA	0.2166	0.5344	0.3439	0.055*	
H5NB	0.1654	0.4517	0.3865	0.055*	
C12	0.26720 (19)	0.4098 (3)	0.4060 (2)	0.0268 (7)	
O1W	0.39648 (14)	0.2667 (2)	−0.01609 (15)	0.0279 (5)	
H1WA	0.4195	0.2642	−0.0607	0.042*	
H1WB	0.4026	0.3377	0.0100	0.042*	
O2W	0.58669 (15)	0.2479 (2)	0.01935 (16)	0.0310 (6)	
H2WA	0.5768	0.2747	−0.0287	0.047*	
H2WB	0.5631	0.1722	0.0319	0.047*	

### Atomic displacement parameters (Å^2^)

**Table:**

	*U*^11^	*U*^22^	*U*^33^	*U*^12^	*U*^13^	*U*^23^
Zr1	0.0173 (2)	0.0148 (2)	0.0169 (2)	0.000	−0.00061 (15)	0.000
O1	0.0241 (12)	0.0232 (11)	0.0261 (11)	−0.0009 (9)	−0.0003 (9)	0.0031 (9)
O2	0.0217 (12)	0.0369 (15)	0.0462 (15)	−0.0031 (11)	0.0017 (11)	0.0095 (12)
O3	0.0213 (11)	0.0233 (11)	0.0232 (11)	−0.0011 (9)	0.0001 (9)	0.0015 (9)
O4	0.0327 (13)	0.0314 (14)	0.0444 (15)	−0.0070 (11)	−0.0008 (12)	0.0144 (12)
O5	0.0280 (11)	0.0211 (11)	0.0216 (11)	−0.0035 (9)	−0.0030 (9)	0.0003 (9)
O6	0.0521 (16)	0.0249 (12)	0.0292 (13)	−0.0054 (11)	−0.0141 (12)	−0.0004 (10)
N1	0.0231 (13)	0.0206 (13)	0.0205 (13)	−0.0005 (10)	0.0002 (10)	−0.0013 (10)
N2	0.0149 (15)	0.0161 (16)	0.0203 (16)	0.000	0.0013 (13)	0.000
C1	0.0236 (15)	0.0212 (14)	0.0228 (15)	0.0026 (12)	−0.0013 (12)	0.0011 (12)
C2	0.0220 (16)	0.0310 (18)	0.0345 (19)	0.0021 (13)	−0.0010 (14)	0.0036 (15)
C3	0.0256 (17)	0.0336 (18)	0.0294 (17)	0.0077 (14)	−0.0024 (14)	0.0045 (15)
C4	0.0311 (17)	0.0233 (16)	0.0276 (16)	0.0032 (13)	−0.0045 (13)	0.0055 (13)
C5	0.0260 (15)	0.0163 (14)	0.0235 (15)	0.0008 (12)	−0.0029 (12)	−0.0002 (12)
C6	0.0192 (14)	0.0226 (15)	0.0235 (15)	−0.0006 (11)	0.0006 (12)	−0.0015 (12)
C7	0.0258 (15)	0.0177 (13)	0.0179 (13)	−0.0029 (12)	−0.0011 (12)	−0.0001 (11)
C8	0.0199 (14)	0.0238 (16)	0.0230 (15)	−0.0014 (12)	0.0018 (11)	−0.0023 (13)
C9	0.0365 (18)	0.0214 (16)	0.0239 (16)	−0.0030 (14)	−0.0006 (13)	−0.0025 (13)
C10	0.040 (3)	0.020 (2)	0.025 (2)	0.000	−0.003 (2)	0.000
C11	0.0244 (15)	0.0240 (16)	0.0235 (15)	−0.0002 (12)	−0.0027 (12)	−0.0022 (12)
N3	0.0240 (14)	0.0321 (16)	0.0375 (17)	−0.0040 (12)	0.0005 (12)	0.0113 (13)
N4	0.0269 (15)	0.0324 (16)	0.0348 (16)	−0.0033 (12)	0.0041 (12)	0.0093 (13)
N5	0.0208 (14)	0.0441 (19)	0.0444 (19)	−0.0014 (13)	0.0018 (13)	0.0160 (15)
C12	0.0275 (17)	0.0300 (17)	0.0228 (16)	−0.0002 (14)	−0.0002 (12)	0.0000 (13)
O1W	0.0299 (12)	0.0258 (12)	0.0280 (12)	−0.0058 (10)	0.0040 (10)	−0.0057 (10)
O2W	0.0336 (13)	0.0273 (12)	0.0323 (13)	−0.0075 (10)	−0.0053 (11)	0.0050 (10)

### Geometric parameters (Å, º)

**Table:**

Zr1—O3	2.203 (2)	C3—H3A	0.9500
Zr1—O3^i^	2.203 (2)	C4—C5	1.384 (4)
Zr1—O1^i^	2.232 (2)	C4—H4A	0.9500
Zr1—O1	2.232 (2)	C5—C7	1.505 (4)
Zr1—O5	2.234 (2)	C8—C9	1.384 (5)
Zr1—O5^i^	2.234 (2)	C8—C11	1.503 (5)
Zr1—N1^i^	2.366 (3)	C9—C10	1.388 (4)
Zr1—N1	2.366 (3)	C9—H9A	0.9500
Zr1—N2	2.378 (4)	C10—C9^i^	1.388 (4)
O1—C6	1.304 (4)	C10—H10A	0.9500
O2—C6	1.222 (4)	N3—C12	1.327 (4)
O3—C7	1.294 (4)	N3—H3NA	0.7713
O4—C7	1.225 (4)	N3—H3NB	0.8647
O5—C11	1.297 (4)	N4—C12	1.329 (4)
O6—C11	1.217 (4)	N4—H4NA	0.8289
N1—C1	1.333 (4)	N4—H4NB	0.7790
N1—C5	1.334 (4)	N5—C12	1.312 (4)
N2—C8	1.336 (4)	N5—H5NA	0.8832
N2—C8^i^	1.336 (4)	N5—H5NB	0.7861
C1—C2	1.393 (5)	O1W—H1WA	0.8370
C1—C6	1.489 (5)	O1W—H1WB	0.8899
C2—C3	1.386 (5)	O2W—H2WA	0.8620
C2—H2A	0.9500	O2W—H2WB	0.9408
C3—C4	1.382 (5)		
			
O3—Zr1—O3^i^	92.30 (12)	N1—C1—C6	113.2 (3)
O3—Zr1—O1^i^	76.29 (8)	C2—C1—C6	124.6 (3)
O3^i^—Zr1—O1^i^	133.53 (8)	C3—C2—C1	117.6 (3)
O3—Zr1—O1	133.53 (8)	C3—C2—H2A	121.2
O3^i^—Zr1—O1	76.29 (8)	C1—C2—H2A	121.2
O1^i^—Zr1—O1	141.94 (12)	C4—C3—C2	120.5 (3)
O3—Zr1—O5	77.18 (8)	C4—C3—H3A	119.8
O3^i^—Zr1—O5	140.67 (8)	C2—C3—H3A	119.8
O1^i^—Zr1—O5	81.18 (9)	C3—C4—C5	117.8 (3)
O1—Zr1—O5	83.89 (9)	C3—C4—H4A	121.1
O3—Zr1—O5^i^	140.67 (8)	C5—C4—H4A	121.1
O3^i^—Zr1—O5^i^	77.18 (8)	N1—C5—C4	122.5 (3)
O1^i^—Zr1—O5^i^	83.89 (9)	N1—C5—C7	111.5 (3)
O1—Zr1—O5^i^	81.18 (9)	C4—C5—C7	126.0 (3)
O5—Zr1—O5^i^	133.07 (12)	O2—C6—O1	124.9 (3)
O3—Zr1—N1^i^	70.31 (8)	O2—C6—C1	121.0 (3)
O3^i^—Zr1—N1^i^	66.58 (8)	O1—C6—C1	114.1 (3)
O1^i^—Zr1—N1^i^	67.17 (9)	O4—C7—O3	124.9 (3)
O1—Zr1—N1^i^	137.20 (9)	O4—C7—C5	121.7 (3)
O5—Zr1—N1^i^	138.75 (9)	O3—C7—C5	113.4 (3)
O5^i^—Zr1—N1^i^	70.75 (9)	N2—C8—C9	121.6 (3)
O3—Zr1—N1	66.58 (8)	N2—C8—C11	112.6 (3)
O3^i^—Zr1—N1	70.31 (8)	C9—C8—C11	125.9 (3)
O1^i^—Zr1—N1	137.20 (9)	C8—C9—C10	118.1 (3)
O1—Zr1—N1	67.17 (9)	C8—C9—H9A	121.0
O5—Zr1—N1	70.75 (9)	C10—C9—H9A	121.0
O5^i^—Zr1—N1	138.75 (9)	C9—C10—C9^i^	120.2 (4)
N1^i^—Zr1—N1	115.98 (13)	C9—C10—H10A	119.9
O3—Zr1—N2	133.85 (6)	C9^i^—C10—H10A	119.9
O3^i^—Zr1—N2	133.85 (6)	O6—C11—O5	125.4 (3)
O1^i^—Zr1—N2	70.97 (6)	O6—C11—C8	121.6 (3)
O1—Zr1—N2	70.97 (6)	O5—C11—C8	113.1 (3)
O5—Zr1—N2	66.54 (6)	C12—N3—H3NA	123.4
O5^i^—Zr1—N2	66.54 (6)	C12—N3—H3NB	115.1
N1^i^—Zr1—N2	122.01 (6)	H3NA—N3—H3NB	120.4
N1—Zr1—N2	122.01 (6)	C12—N4—H4NA	114.6
C6—O1—Zr1	125.7 (2)	C12—N4—H4NB	119.6
C7—O3—Zr1	127.11 (19)	H4NA—N4—H4NB	122.8
C11—O5—Zr1	125.4 (2)	C12—N5—H5NA	119.5
C1—N1—C5	119.5 (3)	C12—N5—H5NB	120.7
C1—N1—Zr1	119.9 (2)	H5NA—N5—H5NB	119.5
C5—N1—Zr1	120.7 (2)	N5—C12—N3	119.5 (3)
C8—N2—C8^i^	120.5 (4)	N5—C12—N4	121.6 (3)
C8—N2—Zr1	119.8 (2)	N3—C12—N4	118.9 (3)
C8^i^—N2—Zr1	119.8 (2)	H1WA—O1W—H1WB	113.4
N1—C1—C2	122.2 (3)	H2WA—O2W—H2WB	114.3
			
O3—Zr1—O1—C6	−5.4 (3)	O3—Zr1—N2—C8^i^	130.96 (17)
O3^i^—Zr1—O1—C6	74.6 (2)	O3^i^—Zr1—N2—C8^i^	−49.04 (17)
O1^i^—Zr1—O1—C6	−138.4 (3)	O1^i^—Zr1—N2—C8^i^	83.72 (17)
O5—Zr1—O1—C6	−71.2 (3)	O1—Zr1—N2—C8^i^	−96.28 (17)
O5^i^—Zr1—O1—C6	153.4 (3)	O5—Zr1—N2—C8^i^	172.17 (17)
N1^i^—Zr1—O1—C6	104.6 (3)	O5^i^—Zr1—N2—C8^i^	−7.83 (17)
N1—Zr1—O1—C6	0.5 (2)	N1^i^—Zr1—N2—C8^i^	38.12 (17)
N2—Zr1—O1—C6	−138.4 (3)	N1—Zr1—N2—C8^i^	−141.88 (17)
O3^i^—Zr1—O3—C7	−59.3 (2)	C5—N1—C1—C2	0.3 (5)
O1^i^—Zr1—O3—C7	166.3 (3)	Zr1—N1—C1—C2	−179.3 (2)
O1—Zr1—O3—C7	13.9 (3)	C5—N1—C1—C6	179.9 (3)
O5—Zr1—O3—C7	82.4 (2)	Zr1—N1—C1—C6	0.4 (4)
O5^i^—Zr1—O3—C7	−131.8 (2)	N1—C1—C2—C3	−1.2 (5)
N1^i^—Zr1—O3—C7	−123.4 (3)	C6—C1—C2—C3	179.2 (3)
N1—Zr1—O3—C7	8.1 (2)	C1—C2—C3—C4	1.1 (5)
N2—Zr1—O3—C7	120.7 (2)	C2—C3—C4—C5	−0.2 (5)
O3—Zr1—O5—C11	166.3 (3)	C1—N1—C5—C4	0.7 (5)
O3^i^—Zr1—O5—C11	−116.0 (3)	Zr1—N1—C5—C4	−179.7 (2)
O1^i^—Zr1—O5—C11	88.4 (3)	C1—N1—C5—C7	−177.9 (3)
O1—Zr1—O5—C11	−56.4 (3)	Zr1—N1—C5—C7	1.7 (3)
O5^i^—Zr1—O5—C11	15.4 (2)	C3—C4—C5—N1	−0.7 (5)
N1^i^—Zr1—O5—C11	127.9 (2)	C3—C4—C5—C7	177.7 (3)
N1—Zr1—O5—C11	−124.3 (3)	Zr1—O1—C6—O2	179.8 (3)
N2—Zr1—O5—C11	15.4 (2)	Zr1—O1—C6—C1	−0.4 (4)
O3—Zr1—N1—C1	175.0 (3)	N1—C1—C6—O2	179.8 (3)
O3^i^—Zr1—N1—C1	−83.4 (2)	C2—C1—C6—O2	−0.6 (5)
O1^i^—Zr1—N1—C1	143.0 (2)	N1—C1—C6—O1	0.0 (4)
O1—Zr1—N1—C1	−0.4 (2)	C2—C1—C6—O1	179.7 (3)
O5—Zr1—N1—C1	91.0 (2)	Zr1—O3—C7—O4	170.7 (2)
O5^i^—Zr1—N1—C1	−43.3 (3)	Zr1—O3—C7—C5	−9.9 (4)
N1^i^—Zr1—N1—C1	−133.3 (3)	N1—C5—C7—O4	−176.1 (3)
N2—Zr1—N1—C1	46.7 (3)	C4—C5—C7—O4	5.4 (5)
O3—Zr1—N1—C5	−4.5 (2)	N1—C5—C7—O3	4.5 (4)
O3^i^—Zr1—N1—C5	97.1 (2)	C4—C5—C7—O3	−174.0 (3)
O1^i^—Zr1—N1—C5	−36.6 (3)	C8^i^—N2—C8—C9	0.9 (2)
O1—Zr1—N1—C5	−180.0 (3)	Zr1—N2—C8—C9	−179.1 (2)
O5—Zr1—N1—C5	−88.5 (2)	C8^i^—N2—C8—C11	−178.4 (3)
O5^i^—Zr1—N1—C5	137.1 (2)	Zr1—N2—C8—C11	1.6 (3)
N1^i^—Zr1—N1—C5	47.2 (2)	N2—C8—C9—C10	−1.8 (5)
N2—Zr1—N1—C5	−132.8 (2)	C11—C8—C9—C10	177.4 (3)
O3—Zr1—N2—C8	−49.04 (17)	C8—C9—C10—C9^i^	0.9 (2)
O3^i^—Zr1—N2—C8	130.96 (17)	Zr1—O5—C11—O6	160.2 (3)
O1^i^—Zr1—N2—C8	−96.28 (17)	Zr1—O5—C11—C8	−19.8 (4)
O1—Zr1—N2—C8	83.72 (17)	N2—C8—C11—O6	−169.6 (3)
O5—Zr1—N2—C8	−7.83 (17)	C9—C8—C11—O6	11.1 (5)
O5^i^—Zr1—N2—C8	172.17 (17)	N2—C8—C11—O5	10.4 (4)
N1^i^—Zr1—N2—C8	−141.88 (17)	C9—C8—C11—O5	−168.9 (3)
N1—Zr1—N2—C8	38.12 (17)		

Symmetry code: (i) −*x*+1, *y*, −*z*+1/2.

### Hydrogen-bond geometry (Å, º)

**Table:**

*D*—H···*A*	*D*—H	H···*A*	*D*···*A*	*D*—H···*A*
N3—H3*NA*···O1	0.77	2.23	3.003 (4)	175
N3—H3*NB*···O2*W*^i^	0.86	2.15	2.930 (4)	150
N4—H4*NA*···O2*W*^i^	0.83	2.04	2.818 (4)	156
N4—H4*NB*···O1*W*^ii^	0.78	2.06	2.834 (4)	175
N5—H5*NA*···O2	0.88	1.95	2.828 (4)	171
N5—H5*NB*···O3^iii^	0.79	2.45	3.143 (4)	148
N5—H5*NB*···O4^iii^	0.79	2.52	3.171 (4)	141
O1*W*—H1*WA*···O3^iv^	0.84	2.33	3.041 (3)	143
O1*W*—H1*WA*···O5^iv^	0.84	2.38	3.076 (3)	140
O1*W*—H1*WB*···O6	0.89	1.87	2.761 (3)	175
O2*W*—H2*WA*···O5^iv^	0.86	2.07	2.909 (3)	165
O2*W*—H2*WA*···O6^iv^	0.86	2.57	3.085 (3)	119
O2*W*—H2*WB*···O4^v^	0.94	1.84	2.745 (3)	160

Symmetry codes: (i) −*x*+1, *y*, −*z*+1/2; (ii) −*x*+1/2, −*y*+1/2, *z*+1/2; (iii) *x*−1/2, *y*−1/2, −*z*+1/2; (iv) −*x*+1, −*y*+1, −*z*; (v) *x*, *y*−1, *z*.
